# Supplementary material for: Anti-Inflammatory, Barrier Maintenance, and Gut Microbiome Modulation Effects of Saccharomyces cerevisiae QHNLD8L1 on DSS-Induced Ulcerative Colitis in Mice
Source: Int J Mol Sci. 2023 Apr 4;24(7):6721. doi: 10.3390/ijms24076721 (PMC10094816; doi:10.3390/ijms24076721)
Supplement: Supplementary file 1 [file ijms-24-06721-s001.zip › ijms-2293700-supplementary.pdf]

Supplementary Information

Table S1: Primer sequences used for RT-qPCR in Caco-2 cells

Table S2: Animal model experimental design

Table S3: Scoring system for disease activity index (DAI)

Table S4: Primer sequences used for RT-qPCR in colitis mice

Figure S1: Effects of *S. boulardii* CNCMI-745, *K. marxianus* QHBYC4L2 and *D. hansenii* QSCLS6L3 on gut microbial functions.

Table S1. Primer sequences used for RT-qPCR in Caco-2 cells.

Genes	Sequence (5'-3')
β-actin	F: CCTTCCCTCCTCAGATCATTGC<br>R: ATACTCCTGCTTGCTGATCCAC
Claudin-1	F: GATGAGGATGGCTGTCATTG<br>R: CCTGACCAAATTCGTACCTG
Occludin	F: CACGCTTGCCTGGGACAGAG<br>R: TCTGTATAGCCTCCGTAGCC
ZO-1	F: CACGCTTGCCTGGGACAGAG<br>R: TCTGTATAGCCTCCGTAGCC

Table S2. Animal model experimental design.

Group	Daily Gavage Treatment (0.2mL)	1-7 Days	8-14 Days
Control	Saline (0.9%)	sterilized water	sterilized water
Model	Saline (0.9%)	sterilized water	DSS water (2.5%)
S. boulardii CNCMI-745	1×10^8 CFU/mL *S. boulardii* CNCMI-745	sterilized water	DSS water (2.5%)
K. marxianus QHBYC4L2	1×10^8 CFU/mL *K. marxianus* QHBYC4L2	sterilized water	DSS water (2.5%)
S. cerevisiae QHNLD8L1	1×10^8 CFU/mL *S. cerevisiae* QHNLD8L1	sterilized water	DSS water (2.5%)
D. hansenii QSCLS6L3	1×10^8 CFU/mL *D. Hansenii* QSCLS6L3	sterilized water	DSS water (2.5%)

Table S3. Scoring system for disease activity index (DAI).

Score	Weight Loss (%)	Stool Consistency	Blood in Feces
0	None	Normal	Negative (no bleeding)
1	1.0~5.0	Loose stools	Negative
2	5.0~10.0	Loose stools	Hemoccult positive (slight)
3	10.0~15.0	Diarrhea (slight)	Hemoccult positive
4	Over 15.0	Diarrhea(Watery diarrhea)	Gross bleeding

Table S4. Primer sequences used for RT-qPCR in colitis mice

Genes	Sequence (5'-3')
β-actin	F: CCTTCCCTCCTCAGATCATTGC<br>R: ATACTCCTGCTTGCTGATCCAC
Claudin-1	F: TGGATGGCTGTCATTGGGG<br>R: ACCTGGCATTGATGGGGGT
Occludin	F: ATAATGGGAGTGAACCCGACG<br>R: CCACGATAATCATGAACCCCA
ZO-1	F: GGGAGGGTCAAATGAAGACA<br>R: GGCATTCCTGCTGGTTACAT

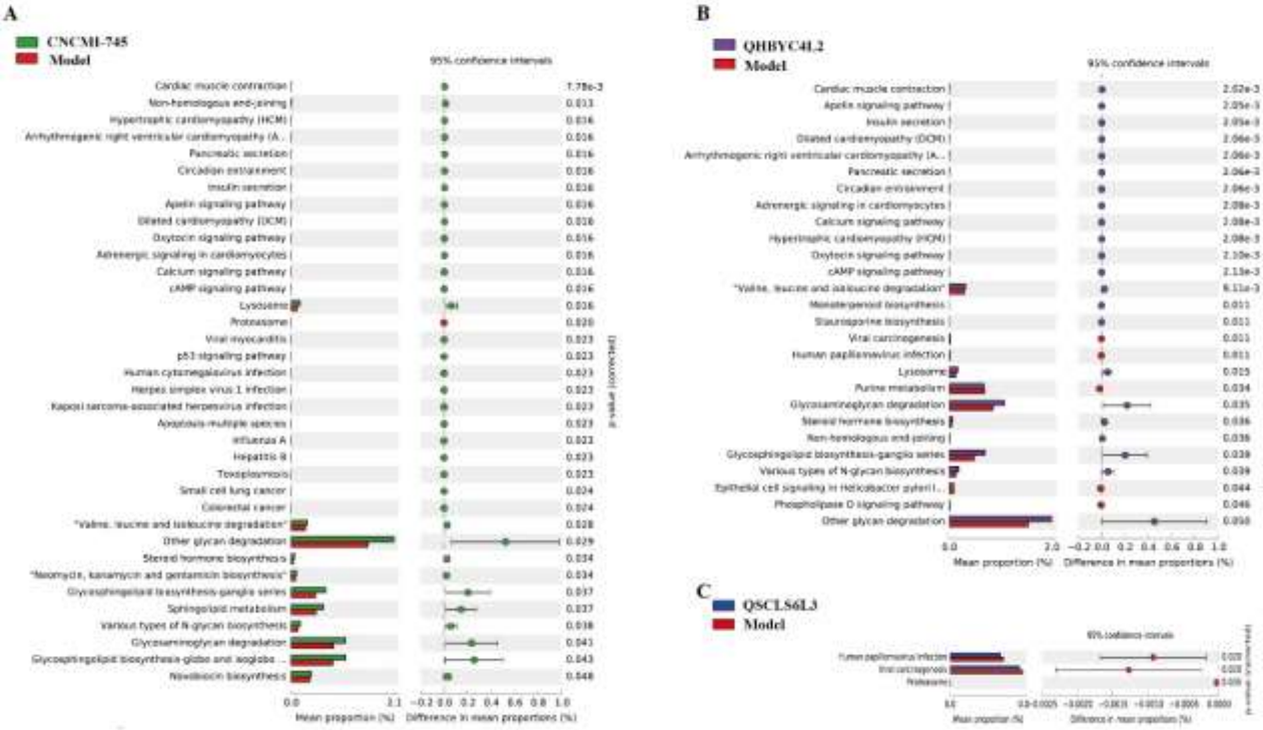

Figure S1. Effects of *S. boulardii* CNCMI-745, *K. marxianus* QHBYC4L2 and *D. hansenii* QSCLS6L3 on gut microbial functions. **(A)** Differences in predicted functions between *S. boulardii* CNCMI-745 and DSS groups. **(B)** Differences in predicted functions between *K. marxianus* QHBYC4L2 and DSS groups. **(C)** Differences in predicted functions between *D. hansenii* QSCLS6L3 and DSS groups.
